# A Severe Gastroenteritis Outbreak of *Salmonella enterica* Serovar Enteritidis Linked to Contaminated Egg Fried Rice, China, 2021

**DOI:** 10.3389/fmicb.2021.779749

**Published:** 2021-11-22

**Authors:** Yaowen Zhang, Kangkang Liu, Zhenbiao Zhang, Sai Tian, Xiong Liu, Hongjuan Qi, Derong Dong, Yong Wang, Meiling Liu, Xinge Li, Yiran Han, Kunpeng Zhu, Hongbo Liu, Chaojie Yang, HONGBO Liu, Xinying Du, Qi Wang, Hui Wang, Mingjuan Yang, Ligui Wang, Hongbin Song, Haiyan Yang, Ying Xiang, Shaofu Qiu

**Affiliations:** ^1^School of Public Health, Zhengzhou University, Zhengzhou, China; ^2^Chinese PLA Center for Disease Control and Prevention, Beijing, China; ^3^College of Veterinary Medicine, Shanxi Agricultural University, Taigu, China

**Keywords:** *Salmonella enterica* serovar enteritidis, outbreak, severe gastroenteritis, whole genome sequencing, virulence gene, phylogenetic analysis

## Abstract

*Salmonella* contamination of eggs and egg shells has been identified as a public health problem worldwide. Here, we reported an outbreak of severe gastrointestinal symptoms caused by *Salmonella enterica* serovar Enteritidis (*S*. enteritidis) in China. We evaluated the outbreak by using epidemiological surveys, routine laboratory testing methods, and whole genome sequencing (WGS). This outbreak occurred in a canteen in Beijing, during March 9–11, 2021, 225 of the 324 diners who have eaten at the canteen showed gastrointestinal symptoms. The outbreak had characteristical epidemiological and clinical features. It caused a very high attack rate (69.4%) in a short incubation time. All patients developed diarrhea and high fever, accompanied by abdominal pain (62.3%), nausea (50.4%), and vomiting (62.7%). The average frequency of diarrhea was 12.4 times/day, and the highest frequency of diarrhea was as high as 50 times/day. The average fever temperature was 39.4°C, and the highest fever temperature was 42°C. Twenty strains of *S*. enteritidis were recovered, including 19 from the patients samples, and one from remained egg fried rice. Antibiotic susceptibility test showed that the 20 outbreak strains all had the same resistance pattern. PFGE results demonstrated that all 20 strains bore completely identical bands. Phylogenetic analysis based on WGS revealed that all 20 outbreak strains were tightly clustered together. So the pathogenic source of this food poisoning incident may was contaminated egg fried rice. Resistance gene analysis showed that the outbreak strains are all multi-drug resistant strains. Virulence gene analysis indicated that these outbreak strains carried a large number of virulence genes, including 2 types of *Salmonella* pathogenicity islands (SPI-1 and SPI-2). Other important virulence genes were also carried by the outbreak strains, such as *pef*ABCD, *rck and shd*A. And the *shd*A gene was not in other strains located in the same evolutionary branch as the outbreak strain. We speculated that this is a significant reason for the serious symptoms of gastroenteritis in this outbreak. This outbreak caused by *S*. enteritidis suggested government should strengthen monitoring of the prevalence of outbreak clone strains, and take measures to mitigate the public health threat posed by contaminated eggs.

## Introduction

World Health Organization (WHO) estimated the global burden of foodborne diseases, the results showed that almost 1 in 10 people fall ill every year from eating contaminated food and 420,000 die as a result (Dewey-Mattia et al., 2018). Salmonellosis is one of the most frequently reported foodborne diseases worldwide. In particular, disease caused by non-typhoid *Salmonella* is a global public health problem, whether in a high-income country or a low-income country (Feasey et al., 2016). Each year, approximately 40,000 *Salmonella* infections are reported to the United States Centers for Disease Control and Prevention (CDCs) (Vaughn et al., 2020). *Salmonella enterica* serovar Enteritidis (*S*. enteritidis) is the predominant *Salmonella* serotype accounting for between 40 and 60% of laboratory-confirmed illnesses of salmonellosis in recent years (Quick et al., 2015). *Salmonella* enteritidis typically cause a self-limiting gastroenteritis with the symptoms of diarrhea, fever, abdominal cramps, and dehydration (Jiang et al., 2020). Salmonellosis is mainly caused by eating eggs and egg products contaminated with *S*. enteritidis (90%) and has become a serious health problem. It has been attributed to this serovar’s unusual ability to colonize ovarian tissue of hens and to be able to present within the contents of intact shell eggs (Chousalkar et al., 2018).

Here we reported a severe gastroenteritis outbreak of *S*. enteritidis linked to contaminated egg fried rice. There were 225 cases of diarrhea and fever in a short period of time in a canteen in Beijing within 3 days. Epidemiological investigations and laboratory tests confirmed that the outbreak was caused by *S*. enteritidis and was related to the undercooked egg fried rice. At present, such a large-scale outbreak with severe clinical symptoms of *S*. enteritidis caused by undercooked eggs is rarely reported (Li et al., 2020). Therefore, we reported the outbreak and examined its molecular characteristics using whole genome sequencing (WGS).

## Materials and Methods

### Outbreak Investigation and Sample Collection

After dinner on March 9, 2021 (18:00), many diarrhea cases occurred in a canteen in Beijing, China. We launched an epidemiological field investigation and concomitant laboratory research in the first time. A suspected salmonellosis case for this outbreak was defined as the onset of diarrhea (>3 times per day), vomiting, or abdominal pain occurring in the staff members who have eaten at the canteen in Beijing during March 9–11, 2021. A confirmed case was that the RT-PCR result is positive for *Salmonella* or our laboratory isolates *Salmonella* from stool samples or rectal swabs (Gidudu et al., 2011).

We collected details of food exposure histories, clinical manifestations, and demographic data. To identify the possible factors that were associated with the outbreak, we conducted a food hygiene survey, focusing on the food preparation process in the base canteen, and collected samples from patients, food and environment. A total of 67 stool specimens and 9 anal swabs from the base staff and canteen service staff were collected, 69 remained food samples, 13 raw eggs, a sample of tap water, and five copies of environmental spreads such as knives were collected. All samples were placed in sterile plastic sample bags, and kept on ice until transported to the laboratory for being tested.

### Pathogen Identification

The collected samples were sent to our laboratory for pathogen isolation and identification. The nucleic acid of samples was extracted with the KingFisher Flex Automatic nucleic acid extractor (Thermo Fisher Scientific, West Sussex, United Kingdom) and LabServ Prefilled Viral NA kit-Flex (Thermo Fisher Scientific, West Sussex, United Kingdom). Twenty-four diarrhea pathogens were screened by using real-time polymerase chain reaction (RT-PCR) with the twenty-four diarrhea pathogens nucleic acid detection kit (BioGerm, Shanghai, China), including *Norovirus Type I*, *Norovirus Type II*, *Campylobacter coli*, *Rotavirus Type A*, *Rotavirus Type B*, *Rotavirus Type C*, *Enteric adenovirus*, *Human Astrovirus*, *sapovirus*, *Vibrio parahaemolyticus*, *Listeria monocytogenes*, *Aeromonas hydrophila*, *Vibrio cholerae*, *Bacillus cereus*, *Yersinia pseudotuberculosis*, *Salmonella*, *Campylobacter jejuni*, *Vibrio fluvialis*, Diarrhea-causing *Escherichia coli*, *Staphylococcus aureus*, *Vibrio mimicus*, *Yersinia enterocolitica*, *Shigella*, *Plesiomonas shigelloides*. Configuration detection system: upstream primer (10 mol/L) 0.625 μL, downstream primer (10 mol/L) 0.625 μL, probe (10 mol/L) 0.5 μL, Enzyme mix 1 μL, RT-QPCR master mix 12.5 μL, water 6.75 μL, DNA template 3 μL. The reaction conditions are as follows: 50°C 30 min, 1 cycle; 95°C 3 min 1 cycle; 95°C 15 s, 55°C 40 s, 40 cycles.

According to Chinese National Standard method (GB 4789.4-2016), the patient’s stool samples were enriched in Selenite Brilliant Green broth (SBG, CHROMagar, Paris, France) at 37°C for 16–22 h. For each food sample, 25 g were transferred to a sterile plastic bag containing 225 mL of Buffered Peptone Water (BPW, Haibo, Qingdao, China) and incubated at 37°C for 18 h, then each pre-enriched homogenate (1 mL) was aseptically added to 10 mL of lactose broth and incubated at 37°C for 24 h. Enriched samples were cultured in *Salmonella-Shigella* agar medium (*SS*, Haibo, Qingdao, China) and MacConkey agar medium (MAC, LandBrigde, Beijing, China) at 37°C for 16–18 h. The typical colony morphology of *Salmonella* on selective *SS* medium is a colorless and transparent colony with a black center. Based on the above criteria, suspected bacteria were selected and inoculated to Luria-Bertani solid agar for second-generation culture, which were incubated at 37°C for 16–18 h, then they were identified using the commercial biochemical test kit (API 20E system; bioMérieux Vitek, Marcy-L’Etoile, France) according to the manufacturer’s instructions. Serogroup identification was performed using the slide agglutination method according to the Kauffmann-White protocol, as described in the manufacturer’s instructions. The isolated *Salmonella* strains were serotyped on a glass slide using a microtiter agglutination test for the O and H antigens (SSI, Copenhagen, Denmark). The 16S rDNA sequence was obtained by PCR amplification and sequencing, and then the obtained sequence was compared with the NCBI database to identify strains.

### Pulsed-Field Gel Electrophoresis

The pulsed field gel electrophoresis (PFGE) of *Salmonella* is carried out in accordance with PulseNet’s 1-day standardized PFGE protocol, with slight modifications (Camarda et al., 2015). *Xba*I (Takara, Dalian, China) was used for restriction endonuclease digestion. The electrophoresis was run on the CHEFER MAPPER variable angle system (Bio-Rad, CA, United States), the parameters were set to 2.16–63.8 s, and the duration was 19 h. The Gel Doc 2000 system (BioRad) was used to capture the images and convert them into TIF format files for further analysis. The collected images were imported into the BioNumerics software (V6.0) database for processing and analysis, and compared with the international standard strain H9812 to calibrate the band position. Cluster analysis uses the arithmetic mean unweighted paired group method (UPGMA).

### Antimicrobial Susceptibility Testing

Antimicrobial susceptibility testing was determined based on the minimum inhibitory concentration (MIC) of each of the 14 common antimicrobial agents, using the Sensititre broth microdilution system and CMV2AGNF plates (Thermo Fisher Scientific, Inc., West Sussex, United Kingdom). The drug sensitivity test plates contained 14 different antibiotics: ceftriaxone (CRO), tetracycline (TET), ceftiofur sodium (XNL), cefoxitin (FOX), gentamicin (GEN), ampicillin (AMP), chloramphenicol (CHL), ciprofloxacin (CIP), meperidine/sulfamethoxazole (SXT), sulfamethoxazole (FIS). *Escherichia coli* ATCC 25922 was used for quality control. Susceptibility tests were interpreted using Clinical and Laboratory Standards Institute (CLSI) guidelines (Melvin et al., 2021).

### Genome Sequencing and Bioinformatics Analysis

The outbreak strains were submitted for whole-genome sequencing. Libraries were prepared using the TruePrepTM DNA Library Prep Kit V2 for Illumina (Vazyme). Using a single “transposase” enzymatic reaction, sample DNA is simultaneously fragmented and tagged with adapters, an optimized, limited-cycle PCR protocol amplifies tagged DNA and adds sequencing indexes. Individual libraries were assessed on the QIAxcel Advanced Automatic nucleic acid analyzer, and then were quantitated using a NanoDrop 2000 (Thermo Scientific, Waltham, MA, United States) spectrophotometer, and verified by agarose gel electrophoresis. At last, the library was sequenced on an Illumina HiSeq Novaseq 6000 platform (Illumina Inc., San Diego, CA, United States) and 150 bp paired-end reads were generated.

Multi-locus sequence typing (MLST) of the outbreak strains was performed according to PUBMLST^1^ (Jolley et al., 2018). We downloaded the complete genome sequences of 513 *S*. enteritidis strains from the NCBI database in April 2021. Core genome single nucleotide polymorphism (SNP) was analyzed using Parsnp (Treangen et al., 2014). The maximum likelihood (ML) method was used to construct phylogenetic tree. And the lineages of the phylogenetic tree were further defined using Bayesian analysis of the population structure (BAPS; version 6.0; Bayesian Statistics Group) (Cheng et al., 2013). In addition, the sequences of outbreak strains and the 513 public strains were analyzed for antimicrobial resistance (AMR) genes and virulence genes. We refer to the relevant literature previously published by our group and set the threshold to 90% (Li et al., 2019). The presence of AMR genes was predicted using the Resistance Gene Identifier (RGI) application of comprehensive antibiotic resistance database (CARD) (Jia et al., 2017), and virulence gene was determined by BLAST against Virulence Factor Database (VFDB) (Liu et al., 2019). Heatmap was drawn using ITOL (v4) (Letunic and Bork, 2019).

## Results

### Epidemiologic Investigation

**FIGURE 1:**
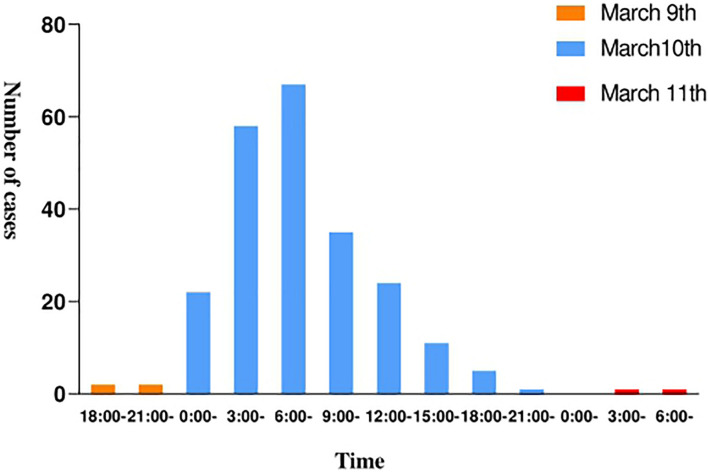
Morbidity curve of a food poisoning incident at a canteen in Beijing.

The epidemic occurred in a canteen in Beijing. All personnel of the base had dinner in the canteen, 225 of the 324 diners had diarrhea, and the overall attack rate is 69.4%. The patients included 223 males and 2 females, and the average age is 24.1 years. The shortest incubation period was 1 h, the longest incubation period was 38.5 h and the average incubation period was 14.3 h (Figure 1). The first case occurred diarrhea symptoms at 19:00 on March 9th, the last case was at 8 o’clock on March 11th. A large number of diarrhea cases occurred after 4 a.m. on the March 10th, accompanied by abdominal pain (62.3%), nausea (50.4%), and vomiting (62.7%). Most patients had diarrhea more than 10 times a day, with the highest diarrhea frequency of 50 times/day. After the symptoms of diarrhea, all cases developed high fever, 81.3% of diarrhea cases developed high fever within 4.5 h, and some cases reached 42°C (Table 1). Food hygiene survey showed that it only took 1 min and 50 s for the egg fried rice to go from frying the raw materials to the finished product. The finished product was placed in a basin, which contained residual raw egg liquid.

**TABLE 1:** Epidemiological characteristics of 225 outbreak cases.

**Characteristics of cases**	**Patients**	**Constituent ratio**
**Gender**		
Male	223	99.1%
Female	2	0.9%
**Symptoms/signs**		
Diarrhea	225	100%
Fever	225	100%
Headache	154	67.5%
General weakness	150	65.8%
Vomiting	143	62.7%
Abdominal pain	142	62.3%
Nausea	115	50.4%
Chills	99	43.4%
Abdominal distension	76	33.3%
Body temperature	–	39.4°C (38–42°C)
Diarrhea frequency	–	12.4 times/day (3–50 times/day)
Incubation period	–	14,3 h (1–38.5 h)

### Laboratory Investigation

Real-time polymerase chain reaction results showed that 42 of the 67 stool samples were positive for *Salmonella* and 8 were weakly positive for *Salmonella*. Five of the nine anal swabs were positive for *Salmonella*. Four of Sixty-nine food samples were positive for *Salmonella*, included two from egg fried rice, one from spicy chicken, and one from preserved egg lean meat porridge. Seven of thirteen eggs were weakly positive for *Salmonella*. Whereas five environmental samples were negative for *Salmonella*, and other intestinal pathogens tests were also negative (Supplementary Table 1). A total of 20 strains were isolated, 16 strains were isolated from base staff, 3 strains were isolated from canteen service staff, and 1 strain was from egg fried rice. Biochemical identification of the isolated strains showed that they were all *Salmonella*. The results of serotyping and 16s rRNA sequencing both showed that 20 outbreak strains were *S*. enteritidis.

### Pulsed-Field Gel Electrophoresis

**FIGURE 2:**
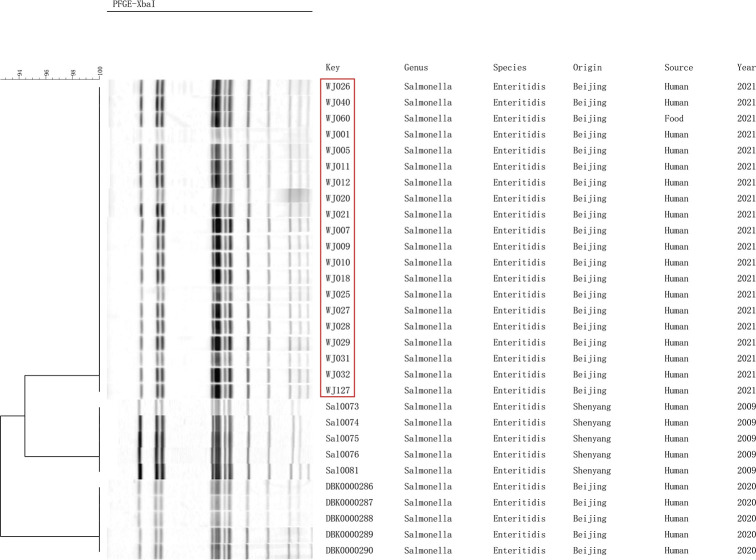
Pulsed-field gel electrophoresis (PFGE) patterns created by digestion with the enzyme *Xba*I.

We performed PFGE cluster analysis of 20 outbreak strains and 10 strains of *S*. enteritidis preserved in our laboratory, and the 20 outbreak *S*. enteritidis strains all had identical PFGE bands. The result demonstrated that it is an outbreak and the egg fried rice is the cause of the outbreak. The PFGE bands of thirty strains formed three branches, and 20 outbreak strains were similar to the strains from Shenzhen exhibiting a similarity of over 90%, indicating that *S*. enteritidis was closely related to each other in different regions of my country (Figure 2).

### Antimicrobial Susceptibility Testing

All 20 outbreak isolates displayed the same multidrug resistance (MDR) profiles (Table 2). The outbreak strains were all resistant to ampicillin (minimum inhibitory concentration, MIC; 32 μg/mL), sulfisoxazole (FIS; 512 μg/mL), nalidixic acid (NAL; 32 μg/mL), tetracycline (TET; 16 μg/mL), and streptomycin (STR; 64 μg/mL), whereas sensitive to ciprofloxacin (CIP; 1 μg/mL), gentamicin (GEN; 4 μg/mL) and ceftriaxone (CRO; 1 μg/mL), azithromycin (AZI; 8 μg/mL), cefoxitin (FOX; 8 μg/mL), ceftiofur (XNL; 2 μg/mL), chloramphenicol (CHL; 8 μg/mL), and sulfamethoxazole (SXT; 2 μg/mL) antibiotics.

**TABLE 2:** Resistance results of 14 antibiotics for 20 outbreak *S*. enteritidis strains.

**Insolation No.**	**Source**	**MICs (μg/ml)^a^**													
		**AUG2**	**AMP**	**AZI**	**FOX**	**XNL**	**CRO**	**CHL**	**CIP**	**GEN**	**NAL**	**STR**	**FIS**	**TET**	**SXT**
WJ001	Human	8	>32	4	2	1	≤0.25	4S	0.25	≤0.25	>32	>64	>256	>32	0.12
WJ005	Human	8	>32	4	2	1	≤0.25	4S	0.12	0.5	>32	>64	>256	>32	≤0.12
WJ007	Human	8	>32	4	1	1	≤0.25	4S	0.25	0.5	>32	>64	>256	>32	0.25
WJ009	Human	8	>32	4	1	1	≤0.25	4S	0.12	≤0.25	>32	>64	>256	>32	0.25
WJ010	Human	8	>32	4	1	1	≤0.25	4S	0.12	0.5	>32	>64	>256	>32	0.25
WJ011	Human	8	>32	8	2	1	≤0.25	8S	0.12	≤0.25	>32	>64	>256	>32	0.25
WJ012	Human	8	>32	4	2	1	≤0.25	4S	0.12	≤0.25	>32	>64	>256	>32	0.25
WJ018	Human	8	>32	4	2	2	≤0.25	4S	0.12	≤0.25	>32	>64	>256	>32	0.25
WJ020	Human	8	>32	4	2	1	≤0.25	8S	0.12	≤0.25	>32	>64	>256	>32	≤0.12
WJ021	Human	8	>32	4	2	1	≤0.25	8S	0.12	≤0.25	>32	>64	>256	>32	0.25
WJ025	Human	8	>32	4	1	1	≤0.25	4S	0.12	0.5	>32	>64	>256	>32	0.25
WJ026	Human	8	>32	4	2	1	≤0.25	8S	0.12	≤0.25	>32	>64	>256	>32	0.25
WJ027	Human	8	>32	4	2	1	≤0.25	4S	0.12	0.5	>32	>64	>256	>32	0.25
WJ028	Human	8	>32	4	1	1	≤0.25	8S	0.12	0.5	>32	>64	>256	>32	0.25
WJ029	Human	8	>32	4	1	2	≤0.25	8S	0.12	0.5	>32	>64	>256	>32	≤0.12
WJ031	Human	8	>32	4	2	1	≤0.25	4S	0.12	0.5	>32	>64	>256	>32	0.25
WJ032	Human	8	>32	4	2	2	≤0.25	8S	0.12	≤0.25	>32	>64	>256	>32	≤0.12
WJ040	Human	8	>32	2	2	1	≤0.25	4S	0.12	≤0.25	>32	>64	>256	>32	≤0.12
WJ060	Food	8	>32	4	2	1	≤0.25	8S	0.12	≤0.25	>32	>64	>256	>32	0.25
WJ127	Human	8	>32	4	2	2	≤0.25	4S	0.12	≤0.25	>32	>64	>256	>32	0.25

*^*a*^CRO, ceftriaxone; TET, tetracycline; XNL, ceftiofur; FOX, cefoxitin; GEN, gentamicin; AMP, ampicillin; CHL, chloramphenicol; CIP, ciprofloxacin; SXT, trimethoprim/sulfamethoxazole; FIS, sulfisoxazole; NAL, nalidixic acid; STR, streptomycin; AZI, azithromycin; AUG2, amoxicillin/clavulanic acid 2:1 ratio.*

### Phylogenetic Analyses

**FIGURE 3:**
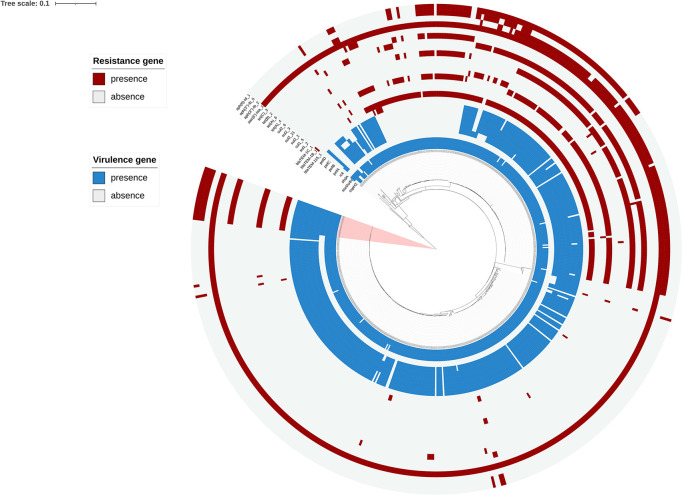
Phylogenetic analysis based on 533 genomes including 20 genomes from the outbreak strains and 513 public genomes. The outbreak strains are light pink areas. Presence and absence of resistance genes were represented by dark red and light gray colors, respectively. Presence and absence of virulence genes is represented by dark blue and light gray colors, respectively.

Phylogenetic analyses showed that the SNP locus difference within the 533 strains was more than 20,000 loci. But the SNP locus difference within the 20 outbreak strains was less than 50 loci, band closely clustered together, which supported that it was indeed an outbreak of *S*. enteritidis infection. The outbreak strains formed a novel small sub-branch separately, which was located inside the main branch of the phylogenetic tree, suggesting that these strains may originated from a single clone (Figure 3). MLST analysis indicated that all 20 strains of the outbreak belonged to sequence type 11 (ST11; Supplementary Table 2).

### Analysis of Drug Resistance and Virulence Genes

We performed AMR genes analysis on 533 *S*. enteritidis strains in Figure 2, which were extracted from WGS analysis. The AMR genes carried by the 20 outbreak strains are exactly the same, including three groups of aminoglycoside resistance genes, namely *aac*(*6′*)*-Iaa_1*, *aph*(*3″*)*-Ib_5*, *aph*(*6*)*-Id_1*, one sulfonamide antibiotic gene, namely *sul2*, and one group of β*-lactamase* gene, namely *bla*_TEM–__1__*B_*__1_ (Supplementary Table 2).

Analysis of the virulence genes of the 533 strains is shown in Supplementary Table 2, where we found that these virulence genes were identical in all outbreaks. All outbreak strains had two types of *Salmonella* pathogenicity islands (*SPI-1* and *SPI-2*), and four types of regulatory function-related genes (*fur*, *phoP*, *phoQ*, and *rpoS*). The identified virulence genes include many genes related to bacterial adhesion and intestinal colonization, such as *csgA-G*, *lpfA-E*, *misL*, *ratB*, *shdA*, and *sinH*, as well as the T3SS gene *sseI/srfH* and bacteriophage encoded *ssspH2*. Interestingly, the plasmidic fimbrial operon *pefABCD* genes, the plasmid mediated gene *rck* and adherence gene *shdA* were detected from outbreak strains, but these genes were missed in the other strains which also located in the main branch (Figure 3).

## Discussion

Here, we reported a food poisoning incident with severe gastroenteritis symptoms. Epidemiological surveys showed that 225 people exhibiting gastrointestinal symptoms all had eaten in the same canteen and presented with symptoms within 3 days. All isolated outbreak strains showed the same antibiotic resistance patterns. PFGE is considered as the “gold standard” for bacterial typing and outbreak detection (Neoh et al., 2019). PFGE results demonstrated that all 20 strains bore completely identical bands, thus confirming this was an outbreak. Previous studies have confirmed that WGS analysis provides better resolution than PFGE in determining the outbreak (Turabelidze et al., 2013). In the phylogenetic analysis, all 20 strains were tightly clustered together, with less than 50 SNP loci found to be inconsistent. These results confirmed that this incident was indeed an outbreak of *S*. enteritidis infection. Moreover, the 20 strains located in a main branch and formed a novel small sub-branch, it may be a newly evolved clone, which deserves our attention and in-depth study.

The epidemiological investigation found that when the egg fried rice was processed for dinner, the shell of the egg was not cleaned before used, and the cooking time of the egg fried rice was too short to guarantee food safety (1 min 50 s). Besides, the processed egg fried rice was placed in a basin, which contained the residual raw materials without cleaning. We speculated that these improper operation practices was the main cause of this outbreak. Twenty strains of *S*. enteritidis were recovered from the patients samples and remained egg fried rice. There are two kinds of ingredients used in egg fried rice, including boiled rice and raw eggs. The RT-PCR results showed that eggs were positive for *Salmonella*. The leftover rice was negative for *Salmonella*. So the contaminated egg fried rice was related to raw eggs carrying *S*. enteritidis. Therefore, this food poisoning incident was successfully traced back to the source, which was contaminated egg fried rice. Although *Salmonella* was not isolated from the eggs, the RT-PCR results showed that the surface of the eggshell was positive for *Salmonella*. So the contaminated egg fried rice may be related to raw eggs carrying *S*. enteritidis. The outbreak had characteristical epidemiological and clinical features. It caused a very high attack rate (69.4%) in a short incubation time, most of the patients are healthy young adults, the average age of who was only 24.1. Moreover, this outbreak caused severe clinical symptoms, and all patients with diarrhea symptoms developed high fever. The average frequency of diarrhea is 12.4 times/day, and the highest frequency of diarrhea was as high as 50 times/day. The average body temperature was 39.4°C, and the highest body temperature was as high as 42°C (Table 1). These epidemiological characteristics are rarely reported in other outbreaks caused by *S*. enteritidis (Inns et al., 2015; Pearce et al., 2018). China is the world’s largest egg producer and a major egg consumer (NBS. China, 2020). Thus, it is important to ensure the quality and safety of eggs. Moreover, the outbreak was related to unsanitary and irregular operations, which attract the government’s attention to strengthen health education.

It is well known that the abuse of antibiotics may lead to the development of multi-drug resistance in Salmonella, leading to increased medical costs and failure of clinical treatment (Li et al., 2020). All 20 isolated strains showed an ASSuT tetra-resistant pattern consistent with the results of resistance genes analysis. All of them encoded the chromosomal aminoglycoside acetyl-transferase gene [*aac*(*6′*)*-Iaa* gene] associated with resistance to STR. The *bla*_TEM_ gene carried by the outbreak strains conferred resistance to AMP. The outbreak isolates co-harbored the *sul2* gene, implicated in the resistance to FIS, and the *tet*(A) gene encoding for Tet resistance. In addition, outbreak strains also showed resistance to NAL. These five antibiotics were the most common antibiotics for which *S*. enterica exhibited resistance, especially in China (Liang et al., 2015; Li et al., 2020). The results of virulence gene analysis showed the isolates presented simultaneously a minimum of 114 virulence-related genes, which are related to the pathogenic potential of *Salmonella* isolates. The majority of important gene-encoded virulence factors are mainly located on pathogenicity islands (SPIs), which are highly conserved in *Salmonella* (Michael et al., 2006). Several genes are important in *Salmonella* virulence, included *inv*A, *spv*C *spa*N, *sip*B, and *sop*B, are associated with the ability to invade the intestinal epithelial cells (Klein et al., 2000; Liebl et al., 2017; Zuo et al., 2020; Krishna and Dhanashree, 2021). The outbreak strains also carried a series of genes associated with adhesion (*lefA-E*), colonization (*Rat*B *and Sin*H) and biofilm formation (*csgA-*G), which promote invasion and survival of strains in unsuitable environments (Desai et al., 2019; Elkenany et al., 2019).

The outbreak strains carried a lot of virulence genes including several special virulence genes. We speculate that it is an important cause of severe gastroenteritis symptoms caused by this outbreak. The 6 virulence genes are *pef*ABCD, *rck*, *and shd*A. *Pef* fimbriae biogenesis depends *pef* operon, located on the virulence plasmid of *Salmonella* (Ledeboer et al., 2006; Hurtado-Escobar et al., 2019). Already reported in the literature, *pef* mutants of *S*. Typhimurium were shown to be attenuated in their ability to form mature biofilms on a chicken intestinal tissue (Ledeboer et al., 2006). And *pef*A was a gene encoding major fimbrial subunit, which was the most-segregative virulence factor (Alshalchi et al., 2017). Rck protein belongs to the Ail/Lom protein family that consists of several bacterial or phage outer membrane proteins (OMP), these proteins are involved in the expression of pathogen virulence and *rck* play a key role in invasion of different host cells (Rosselin et al., 2010; Mambu et al., 2017). *ShdA* gene is unique in the 20 outbreak strains, while deleted in the other 513 strains. The role of *shdA* in *S*. typhimurium is mainly participated in adherence/invasion of fibronectin-producing cells (Dos et al., 2021), and *shdA*-mediated binding of the extracellular matrix contributes to persistent intestinal carriage (Kingsley et al., 2000; Urrutia et al., 2014). Interestingly, the study found that *shd*A gene were only carried in outbreak strains, but not in other strains located in the same evolutionary branch as the outbreak strain. However, the mechanism effect of *shdA* in *S*. enterica has not been reported before, and this will be the focus of our next exploration.

## Conclusion

We described an outbreak in China caused by *S*. enteritidis, which resulted in more than 200 people to develop severe gastroenteritis symptoms in a short period of time. The outbreak was mainly caused by contaminated egg fried rice, which is a kind of delicacy with Chinese characteristics. WGS analysis results showed that 20 isolated strains carried consistent resistance genes and multiple virulence factors. Moreover, several significant virulence genes were carried in outbreak strains, included *pefABCD*, *rck* and *shdA*. We speculate that this is an important reason for the serious symptoms of gastroenteritis in this epidemic. The prevalence of the outbreak clone strains should be monitored. The results of this study justified that it is necessary to strengthen the education of food processors and consumers about the risk of cross-contamination of raw eggs and food, and to improve hygiene measures to prevent diseases caused by *S*. enteritidis.

## Data Availability Statement

The datasets presented in this study can be found in online repositories. The names of the repository/repositories and accession number(s) can be found below: NCBI (accession: PRJNA764371).

## Author Contributions

YZ wrote the main manuscript and participated in all experiments. SQ, HY, and YX designed the study and reviewed the manuscript. HQ, DD, XLiu, and YW conducted epidemiological investigation on this outbreak. HL (9th author), CY, HL (11th author), XD, QW, HW, MY participated in data collection. ST, ML, XLi, and YH participated in experiments. ZZ, KL, KZ, LW, and HS contributed to the bioinformatics data analysis. SQ, HY, and YX gave final approval of the version to be submitted. All authors made substantial contributions to preparation and submission of manuscript.

## Conflict of Interest

The authors declare that the research was conducted in the absence of any commercial or financial relationships that could be construed as a potential conflict of interest.

## Publisher’s Note

All claims expressed in this article are solely those of the authors and do not necessarily represent those of their affiliated organizations, or those of the publisher, the editors and the reviewers. Any product that may be evaluated in this article, or claim that may be made by its manufacturer, is not guaranteed or endorsed by the publisher.

## Footnotes

1
https://pubmlst.org/

## Supplementary Material

Supplementary Figure 1 The vertical version of phylogenetic analysis based on 533 genomes including 20 genomes from the outbreak strains and 513 public genomes. The outbreak strains are light pink areas. Presence and absence of resistance genes were represented by dark red and light gray colors, respectively. Presence and absence of virulence genes were represented by dark blue and light gray colors, respectively.
