# Corrigendum: A Severe Gastroenteritis Outbreak of *Salmonella enterica* Serovar Enteritidis Linked to Contaminated Egg Fried Rice, China, 2021

**DOI:** 10.3389/fmicb.2022.847423

**Published:** 2022-02-10

**Authors:** Yaowen Zhang, Kangkang Liu, Zhenbiao Zhang, Sai Tian, Xiong Liu, Hongjuan Qi, Derong Dong, Yong Wang, Meiling Liu, Xinge Li, Yiran Han, Kunpeng Zhu, Hongbo Liu, Chaojie Yang, HONGBO Liu, Xinying Du, Qi Wang, Hui Wang, Mingjuan Yang, Ligui Wang, Hongbin Song, Haiyan Yang, Ying Xiang, Shaofu Qiu

**Affiliations:** ^1^School of Public Health, Zhengzhou University, Zhengzhou, China; ^2^Chinese PLA Center for Disease Control and Prevention, Beijing, China; ^3^College of Veterinary Medicine, Shanxi Agricultural University, Taigu, China

**Keywords:** *Salmonella enterica* serovar enteritidis, outbreak, severe gastroenteritis, whole genome sequencing, virulence gene, phylogenetic analysis

**Additional Affiliation(s)**

In the published article, there was an error regarding the affiliation for ^**^Shaofu Qiu^**^. As well as having affiliation ^**^2 ^**^, they should also have ^**^School of Public Health, Zhengzhou University, Zhengzhou, China ^**^. We apologize for this error and state that this does not change the scientific conclusions of the article in any way. The original article has been updated.

**Addition of Authors**

^**^Xiong Liu, Hongjuan Qi, Derong Dong, Yong Wang^**^ was not included as authors in the published article. The corrected Author Contributions Statement appears below.

## Author Contributions

YZ wrote the main manuscript and participated in all experiments. SQ, HY, and YX designed the study and reviewed the manuscript. HQ, DD, XLiu, and YW conducted epidemiological investigation on this outbreak. HL (9th author), CY, HL (11th author), XD, QW, HW, MY participated in data collection. ST, ML, XLi, and YH participated in experiments. ZZ, KL, KZ, LW, and HS contributed to the bioinformatics data analysis. SQ, HY, and YX gave final approval of the version to be submitted. All authors made substantial contributions to preparation and submission of manuscript.

We apologize for this error and state that this does not change the scientific conclusions of the article in any way. The original article has been updated.

## Publisher's Note

All claims expressed in this article are solely those of the authors and do not necessarily represent those of their affiliated organizations, or those of the publisher, the editors and the reviewers. Any product that may be evaluated in this article, or claim that may be made by its manufacturer, is not guaranteed or endorsed by the publisher.

